# Hierarchically Porous Fe−N−C Single‐Atom Catalysts via Ionothermal Synthesis for Oxygen Reduction Reaction

**DOI:** 10.1002/cssc.202401332

**Published:** 2024-10-23

**Authors:** Kaarel Kisand, Ave Sarapuu, John C. Douglin, Arvo Kikas, Maike Käärik, Jekaterina Kozlova, Jaan Aruväli, Alexey Treshchalov, Jaan Leis, Vambola Kisand, Kaupo Kukli, Dario R. Dekel, Kaido Tammeveski

**Affiliations:** ^1^ Institute of Chemistry University of Tartu Ravila 14a Tartu 50411 Estonia; ^2^ Institute of Physics University of Tartu W. Ostwald Str. 1 Tartu 50411 Estonia; ^3^ Institute of Ecology and Earth Sciences University of Tartu Vanemuise 46 Tartu 51014 Estonia; ^4^ The Wolfson Department of Chemical Engineering Technion-Israel Institute of Technology Haifa 3200003 Israel; ^5^ The Nancy & Stephen Grand Technion Energy Program (GTEP) Technion-Israel Institute of Technology Haifa 3200003 Israel

**Keywords:** Oxygen reduction reaction, PGM-free catalysts, Single-atom catalysts, M−N−C catalysts, Anion exchange membrane fuel cells

## Abstract

Platinum group metal (PGM)‐free electrocatalysts have emerged as promising alternatives to replace Pt for the oxygen reduction reaction (ORR) in anion exchange membrane fuel cells (AEMFCs). However, traditional synthesis methods limit the single‐atom site density due to metal agglomeration at higher temperatures. This work explores the preparation of hierarchically porous atomically dispersed electrocatalysts for the ORR. The materials were prepared via ionothermal synthesis, where magnesium nitrate was used to prepare hierarchically porous carbon materials. The in‐situ formed Mg‐N_x_ sites were trans‐metalated to yield ORR‐active Fe‐N_x_ sites. The resulting carbon‐based catalysts displayed excellent electrocatalytic activity, attributed to the atomically dispersed Fe‐N_x_ active sites and high meso‐ and macroporosity that enhanced the mass transport and exposed more accessible active sites.

## Graphical abstract

This study explores the preparation of catalysts with atomically dispersed active sites for the ORR. Traditional synthesis methods limit single‐atom site density in M−N−C catalysts, due to metal agglomeration. We use a novel ionothermal approach to create the catalysts, resulting in hierarchical porosity and imprinted Mg‐N_x_ sites, which are then trans‐metalated to Fe‐N_x_ sites. The resulting catalysts show excellent ORR activity and AEMFC results.

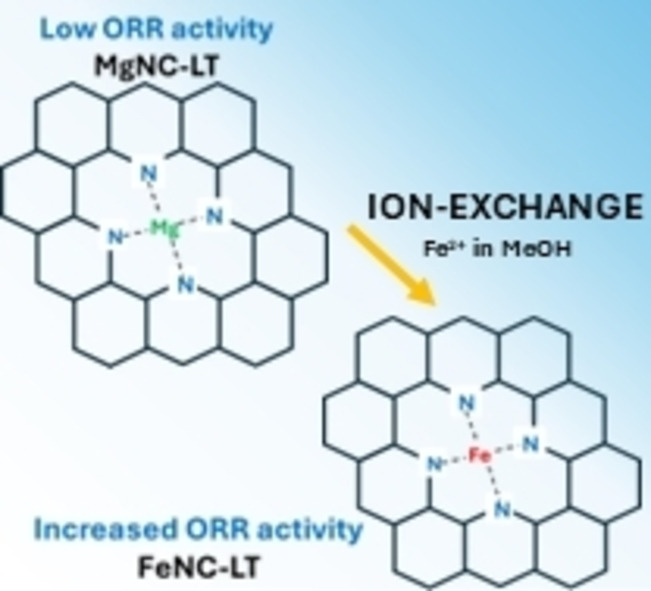

## Introduction

The widespread adoption of proton exchange membrane fuel cells (PEMFCs) faces challenges due to constraints associated with the cathode and the oxygen reduction reaction (ORR) in low‐pH environments.[^1^, ^2^] Namely, the sluggish kinetics of the ORR and durability concerns in highly corrosive acidic conditions require platinum‐group metal (PGM) catalysts.[^3^, ^4^] Due to this, significant research efforts have been made towards anion exchange membrane fuel cells (AEMFCs) that make use of PGM‐free electrocatalysts.[^5^, ^6^, ^7^, ^8^, ^9^, ^10^, ^11^]

The inherent electrocatalytic activity of a PGM‐free catalyst is tied to its surface moieties. While there is no unanimous agreement on which specific centers contribute to the ORR activity, transition metal‐nitrogen‐coordinated (M‐N_x_) sites are generally considered the primary active sites responsible for electrocatalytic activity in PGM‐free materials.[^12^, ^13^, ^14^, ^15^, ^16^] In the past few years, the advancement in synthesis methodologies have resulted in the development of transition metal‐nitrogen‐carbon (M−N−C) catalysts for ORR electrocatalysis, mostly containing M‐N_x_ sites with minimal metal nanoparticles.[^17^, ^18^, ^19^, ^20^, ^21^, ^22^] Still, the primary challenge associated with PGM‐free catalyst materials lies in their low site density (SD). Despite notable progress in synthesis techniques, such as employing metal‐organic framework (MOF) precursors to restrict ion mobility and prevent metal agglomeration, achieving a high SD remains a challenge.[^23^, ^24^, ^25^, ^26^, ^27^] This difficulty arises because higher concentrations of metal precursors lead to condensation resulting in the formation of metal nanoparticles above 600 °C.[^28^, ^29^, ^30^, ^31^, ^32^, ^33^] To address the challenges associated with traditional high‐temperature synthesis, an emerging approach proposed by Fellinger et al. decouples the formation of carbon matrices from the active site creation.^[28]^ The active site imprinting strategy leverages specific imprinting metals (such as Zn or Mg) that promote the formation of the desired nitrogen coordination sites. The approach involves initially preparing a Mg‐NC or Zn‐NC material, followed by a subsequent low‐temperature coordination step, during which the Mg (or Zn) ion in the coordinated site is replaced (trans‐metalated) by an electrocatalytically active element, such as iron.[^28^, ^34^] Due to the temperatures commonly employed in the pyrolytic carbon preparation, the reduction of Mg^2+^ and Zn^2+^ ions is not thermodynamically favorable. This strategy enables the creation of M−N−C catalysts with significantly higher density of M–N_x_ sites by preventing undesired side reactions.[^34^, ^35^]

However, not all M‐N_x_ moieties can be considered active sites, because the sites buried deep in the carbon microstructure are not effectively participating in the ORR process.^[36]^ The hierarchical structure of the carbon will lead to greater utilization of these sites, as only sites at the triple phase boundary (TPB) can participate in the ORR at the fuel cell cathode.[^18^, ^37^, ^38^] Carbon materials with high surface area and hierarchical porosity can be made by deliberate structuring at the micro‐, meso‐ and macroscale during their preparation through a variety of techniques. The most common methods for producing additional porosity are physical activation by using reactive gases such as steam and CO_2_[^39^, ^40^] and chemical activation.^[41]^ Although these methods result in a high surface area, they create mainly microporous carbon materials. Usually, synthetic methods based on using templates are employed to create the desired meso‐ and macroporous structure.[^42^, ^43^, ^44^] Silica nanoparticles are commonly used during thermal treatment to prepare carbon materials with controlled porous structure.[^44^, ^45^] In this process, the template remains thermodynamically stable at high temperatures and the template particles are removed after pyrolysis via chemical etching, resulting in carbon materials with a controlled structure. However, the use of concentrated solutions of HF or NaOH for template removal poses a significant limitation due to their toxicity, hindering the scalability and raising environmental concerns.^[42]^ In addition, the recycling of silica‐based templates has not been tried due to the expected high cost of the procedure. Another approach for achieving hierarchical porosity in carbon materials is through ionothermal synthesis.[^46^, ^47^] Here, a low‐melting point Mg salt is used as the reaction media during pyrolysis. The porous carbon nanostructure is first created through the molten salt droplets and secondary porosity is obtained after the in‐situ formed MgO nanoparticles are removed via acid‐treatment. The recyclability of Mg has been experimentally confirmed in the work of Morishita et al.^[43]^ The interaction between the precursor phase (organic material) and the molten salt plays a crucial role in determining the resulting porosity. Additionally, the selection of salt can significantly impact the nanostructure and within certain limits, changing the ratio of salt to organic precursor can modify the resulting carbon structure.^[48]^

In this work we used ionothermal synthesis, utilizing Honeyol (mixture of alkylresorcinols) and Mg(NO_3_)_2_ to prepare carbon‐based catalysts with a hierarchical structure, high surface area and imprinted Mg‐N_x_ sites. Subsequent acid leaching of the in‐situ formed MgO nanoparticles produces hierarchically porous carbon materials. The resulting morphology is promising for exposing active sites and enhancing reactant mass transfer during electrocatalysis. To create ORR‐active atomically dispersed Fe‐N_x_ sites, the Mg was replaced with Fe via an ion‐exchange method. The catalysts were thoroughly characterized and the ORR electrocatalytic activity of the prepared catalysts was evaluated via the rotating disk electrode (RDE) method to gain insight into the structure‐activity relationships. Finally, their performance as cathode catalysts in an AEMFC was evaluated.

## Results and Discussion

### Structural Characterization

**Figure 1:**
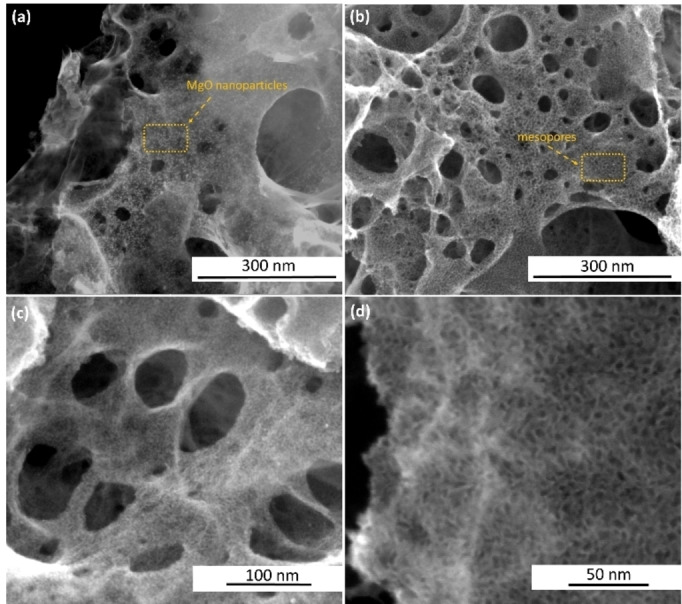
SEM images of (a) MgO@NC‐LT, showing the MgO nanoparticles and (b,c and d) NC‐LT materials, showing the mesopores created after the acid treatment.

In the ionothermal synthesis, Honeyol is partially or completely dissolved within the salt melt. Mg(NO_3_)_2_ ⋅ 6H_2_O was chosen as the precursor since it has a low melting point (88.9 °C), which is below the onset of carbonization of organic species and can thus act as an ionothermal liquid reaction medium. While a variety of magnesium salts can be used, we specifically selected the nitrate for the secondary purpose of introducing nitrogen moieties into the carbonizing phase. During pyrolysis, the generation of pores occurs through two main effects. Firstly, the mostly macroporous structure is created by the liquid salt droplets inside the carbonizing phase.^[48]^ The scanning electron microscopy (SEM) images of the materials display a hierarchically porous structure (Figure 1), where both macropores and larger mesopores can be seen. The secondary porosity is created when the carbonizing phase encircles the in‐situ formed MgO nanoparticles (so‐called hard template).[^43^, ^49^] The SEM image of MgO@NC‐LT displays numerous MgO nanoparticles that are evenly embedded in the carbon structure (Figure 1a).

**Figure 2:**
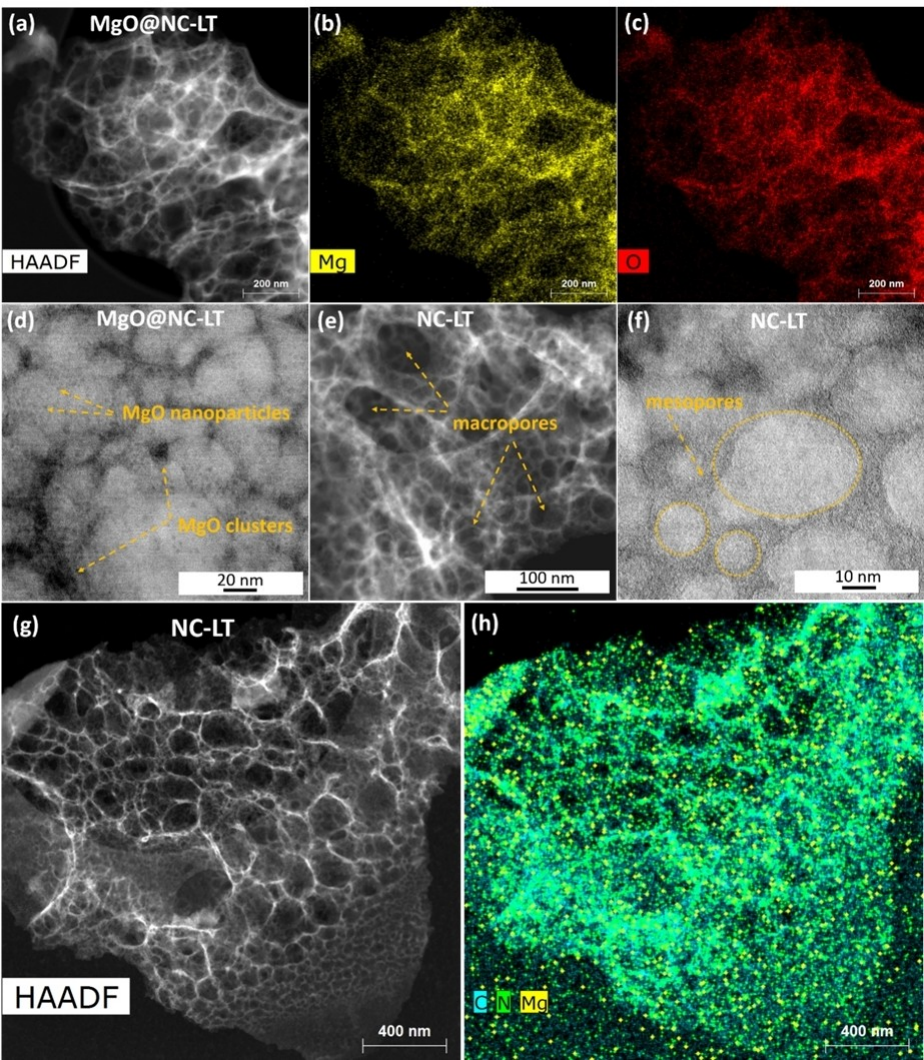
(a) HAADF‐STEM image of MgO@NC‐LT and (b, c) corresponding EDX maps of Mg and O elements. (d and f) BF‐STEM images, (e and g) HAADF‐STEM images of NC‐LT and (h) the overlay of EDX maps of C, N and Mg elements.

The high‐angle annular dark‐field scanning transmission electron microscopy (HAADF‐STEM) image of MgO@NC‐LT and elemental mapping by energy‐dispersive X‐ray spectroscopy (EDX) (Figure 2a–c) reveal an even distribution of Mg across the carbon structure with no large MgO agglomerates observed. BF‐STEM images (Figure S1d and e) reveal the presence of small spherical nanoparticles embedded in the carbon matrix. Once these nanoparticles are removed via acid‐washing, additional mesopores are created within the carbon material as can be seen from Figure 1b. These pores seem to range in size from approximately 3 to 20 nm, corresponding to the size of the MgO nanoparticles, with the larger mesopores formed from aggregated clusters (Figure 2d). The STEM images (Figure 2e and f) provide a more detailed look into the carbon structure after removal of MgO nanoparticles. The hierarchically porous morphology of NC‐LT is promising for exposing a large number of active sites and enhancing the mass transport of reactants in the catalyst layer.[^37^, ^50^]

**Figure 3:**
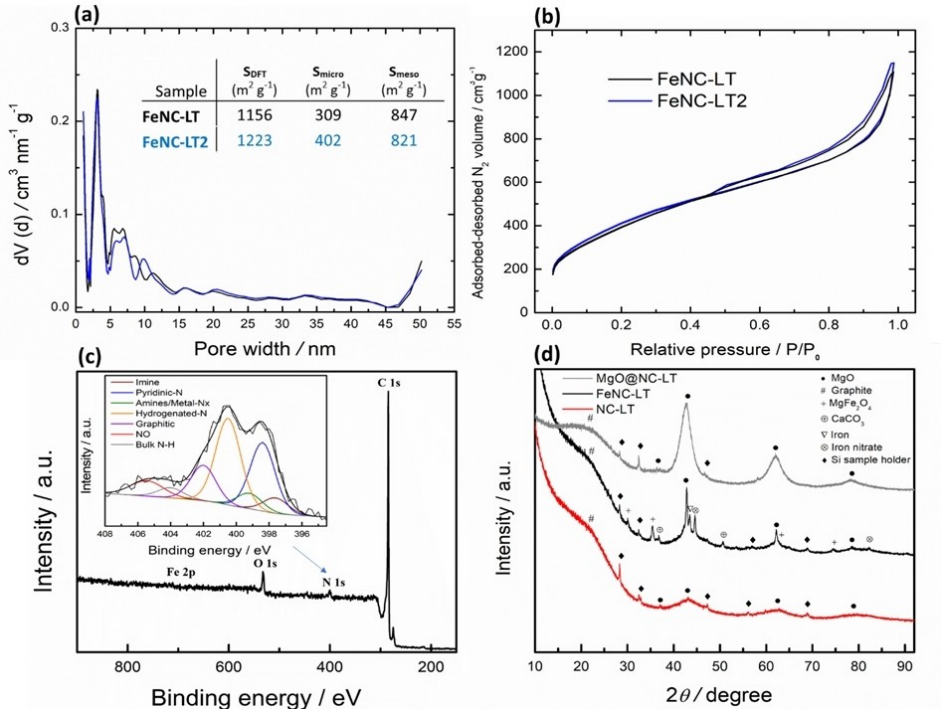
(a) Pore size distribution of the prepared catalysts with the *S*_DFT_ values shown in the inset; (b) N_2_ isotherms of catalyst materials; (c) XPS survey spectrum for FeNC‐LT with the inset showing detailed XPS N 1s spectra; and (d) XRD patterns for various materials.

**Figure 4:**
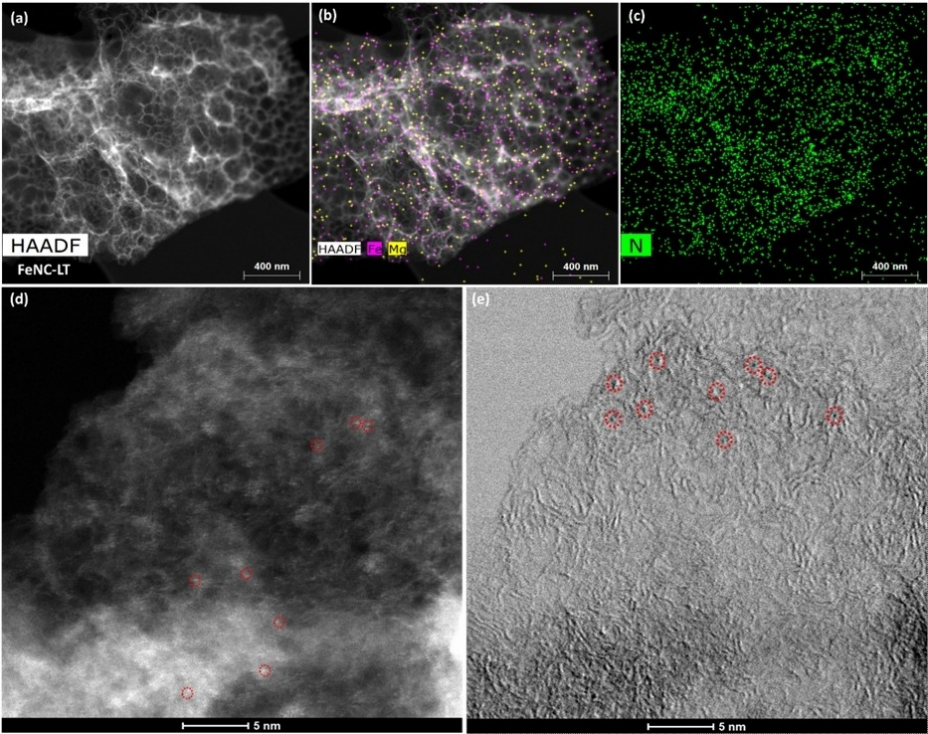
(a) HAADF‐STEM image of FeNC‐LT and (b, c) overlays of EDX maps of Fe, Mg, and N elements. (d, e) HAADF‐STEM and BF images of FeNC‐LT with some of the atomically dispersed sites circled in red.

The nitrogen adsorption isotherms of the catalysts and the associated pore size distribution (PSD) are shown in Figure 3. The isotherms display a steep rise at low pressure, indicating some microporosity in the carbon materials, which is caused by the rapidly evolved gases during the decomposition of the nitrate species (Figure 3b). A slope can be seen forming at higher relative pressures, indicating the filling of mesopores. The presence of macroporosity can be assumed from the PSD graph, however, due to the upper limit of detection for this method (50 nm for N_2_ adsorption) more precise information about the PSD at larger pore sizes cannot be determined.^[51]^ However, the presence of macropores and large mesopores that are introduced into the carbonizing precursors by molten salt droplets, was confirmed in both SEM and STEM measurements. Comparing the PSDs of FeNC‐LT and FeNC‐LT2, we observe an increase in microporosity and a slight decrease in mesoporosity. The higher amount of the templating salt in FeNC‐LT2 leads to a broadening of mesopores, likely caused by more extensive particle agglomeration. This is further evidenced by a slight decrease in mesoporous specific surface area. It is apparent that the porosity can be controlled by varying the organic precursor to salt template ratio. The microporosity increase in the FeNC‐LT2 material results from a larger amount of gases evolved during the decomposition of the Mg(NO_3_)_2_.HAADF‐STEM and the EDX mapping overlay (Figure 2g and h) evidence the successful removal of most of the MgO nanoparticles and the formation of mesopores. However, some MgO is apparently left in the material even after acid treatment as evidenced by the X‐ray diffraction (XRD) pattern for NC‐LT (Figure 3d), where a MgO phase (PDF‐00‐045‐0946) with an average crystallite size of around 4 nm was detected. This same phase is also observed in the non‐acid washed material MgO@NC‐LT in much greater quantities. Nitrogen is evenly distributed over the entire carbon structure, indicating the successful introduction of the dopant element. Around 2 wt.% of Mg can be detected in the NC‐LT material after the acid washing (Table S2). Additionally, we performed Raman spectroscopy measurements with the two catalysts (Figure S3), where very broad D and G peaks were observed for both materials indicating a rather amorphous carbon structure. The ratio of peak intensities (*I*_D_ */I*_G_ ratio) was determined to be 0.91.The X‐ray photoelectron spectroscopy (XPS) analysis of the material was conducted to obtain more detailed information about the moieties present on the surface. XPS displays peaks at 285, 400, and 532 eV, corresponding to binding energies of C 1s, N 1s, and O 1s (Figure 3b and S4a). Around 3 at. % of nitrogen is detected on the surface of the NC‐LT material. A closer look at the high‐resolution of N 1s spectra reveals that hydrogenated or pyrrolic nitrogen is the most common N moiety. This supports the findings from Menga et al.^[28]^ that mainly tetrapyrrolic M‐N_x_ sites are formed during the pyrolysis process in the presence of Zn or Mg salts, as minimal carbothermal reduction occurs. This is supported by the C 1s spectra of NC‐LT and FeNC‐LT (Figure S4b and c), where most of the carbon is sp^3^ with some sp^2^ (characteristic of highly defective and disordered carbon material) or present in various oxygen‐containing moieties, while the carbide peak is very faintly detected. For the FeNC‐LT material, obtained after the trans‐metalation procedure and subsequent pyrolysis, the pyrrolic N remains the most common moiety. As the pyrrolic nitrogen peak remains unaltered following the heat treatment, the pyrrolic Fe−N bonds seem to be robust at temperatures up to 800 °C, without undergoing decomposition. However, comparison of N 1s spectra of NC‐LT (Figure S4a, inset) and FeNC‐LT (Figure 3c, inset) reveals that additional pyridinic nitrogen sites are created in the latter. Formation of pyridinic N sites requires graphitization of the carbon structure (occurring at above 1300 °C), but it is well known that iron can act as a catalyst for graphitization at lower temperatures. Some weakly crystalline iron‐containing nanoparticles (most likely in oxide, chloride or hydroxide form) are present in the material after the trans‐metalation procedure, as the acid‐treatment did not successfully remove all the adsorbed iron‐containing species (Figure S5), which then agglomerate during the 2^nd^ pyrolysis. The iron‐containing nanoparticles can decompose and release Fe atoms, which can then coordinate with nitrogen, leading to increased density of Fe‐N_x_ sites.^[52]^ This was also observed in the work of Menga et al., who observed 90 % reduction of oxidized iron by Mössbauer spectroscopy after the second pyrolysis.^[28]^ From the HAADF‐STEM image and corresponding EDX maps for FeNC‐LT (Figure 4), we observe no change in the morphology of the material and an even distribution of Fe and Mg, while no large agglomerated metallic iron or carbide nanoparticles can be observed, indicating that Fe is mainly atomically dispersed and likely coordinated to nitrogen. Higher magnification HAADF and BF images (Figure 4d,e and S6) display the M‐N_x_ sites present in the material, while small clusters of presumably iron or magnesium can be observed in some areas (Figure S2b). The nature and elemental composition of these clusters as well as whether they are bound to neighboring C/N atoms or not, remains unknown.

The synergistic effects of nanoparticles together with single atoms in M−N−C catalysts is well‐supported by literature, especially in alkaline media.[^53^, ^54^, ^55^] Although a scientific consensus as to the synergetic effect and which of active sites contribute to the ORR has not been reached despite extensive research. From the XRD pattern of FeNC‐LT we can observe some crystalline iron phases such as metallic iron (PDF‐04‐002‐3692) and iron nitrate (PDF‐04‐021‐9691). In addition to the MgO phase (PDF‐00‐045‐0946) also present in NC‐LT, a more agglomerated phase (PDF‐04‐008‐3504), with an average crystallite size of 38 nm (Figure 3d) was also observed, likely forming during the second pyrolysis. In addition, some MgFe_2_O_4_ (PDF‐04‐012‐0947) XRD peaks were observed. The carbon material is highly disordered, consistent with the XPS high‐resolution C 1s spectra. Peaks indexed to CaCO_3_ phase (PDF‐01‐088‐8687) can also be detected in the FeNC‐LT material, most likely due to the contamination. Since Mg can still be detected in the FeNC‐LT material, it would suggest that the trans‐metalation procedure was not able to convert all of the Mg‐N_x_ sites. If the site is located deep in the micropores of the carbon matrix, it is unlikely that the Fe ions can reach the sites during the ion exchange process. We presume that further research is needed to find the right conditions for the ion exchange step to ensure the highest possible conversion of Mg‐N_x_ sites. The presence of 2.1 wt.% of iron in FeNC‐LT is verified through microwave plasma atomic emission spectroscopy (MP‐AES) analysis, while no Fe can be detected in NC‐LT material (Table S2). Around 2 wt.% of Mg can be detected in both NC‐LT and FeNC‐LT, however the exact determination of the efficiency of Mg‐to‐Fe conversion is problematic, since some of the Mg is in the form of MgO and MgFe_2_O_4_ nanoparticles according to the XRD analysis. The interpretation is further convoluted if the sample inhomogeneity is considered.

### Electrochemical Characterization

**Figure 5:**
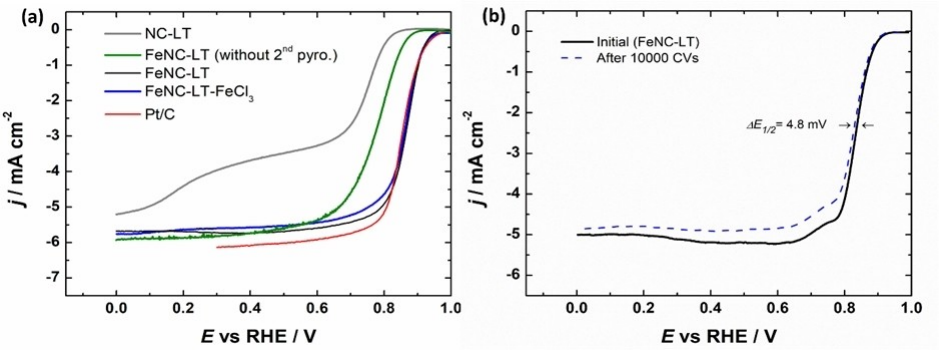
(a) Comparison of RDE voltammetry curves for ORR on various PGM‐free catalysts and commercial Pt/C in O_2_‐saturated 0.1 M KOH solution at 1900 rpm, (b) RDE voltammetry curves for O_2_ reduction before and after repetitive potential cycles for the FeNC‐LT catalyst.

The electrocatalytic performance of the catalysts was initially assessed through rotating disc electrode (RDE) testing in O_2_‐saturated 0.1 M KOH (Figure 5a and S7). The NC‐LT catalyst exhibited low ORR activity characterized by a large overpotential. This catalyst was prepared without the ion‐exchange step and since it does not contain any Fe‐N_x_ sites, the electrocatalytic activity originates from the nitrogen moieties in the carbon matrix. These sites are generally considered to be ORR‐active, however, they catalyze the ORR mainly via the 2‐electron pathway.[^56^, ^57^, ^58^] Following low‐temperature ion exchange from Mg‐N_x_ to Fe‐N_x_ and the acid treatment, the ORR electrocatalytic activity of the catalyst material is significantly increased, exhibiting a half‐wave potential (*E*_1/2_) of 0.77 V. Subsequent high‐temperature treatment further enhances the catalyst′s activity, attributed to the increased density of active sites that are formed during the pyrolysis when agglomerated iron particles decompose and coordinate with either pyridinic nitrogen or left‐over Mg‐N_x_ sites. The FeNC‐LT catalyst reaches an impressive *E*_1/2_ value of 0.87 V, which is comparable to the benchmark Pt/C catalyst. Interestingly, the catalyst prepared with a larger Mg(NO_3_)_2_‐to‐Honeyol mass ratio (FeNC‐LT2) showed diminished ORR activity (Figure S7). Since the higher Mg‐precursor amount leads to greater agglomeration of the MgO particles, the mesoporosity of this material was reduced as evidenced by the N_2_ adsorption/desorption measurements, possibly limiting the number of accessible active sites in the catalyst and leading to an increase in the overpotential.

As was mentioned previously, since not all of the Mg in the material was exchanged to Fe, we additionally tried preparing a catalyst using the Fe^3+^ ions during the ion exchange. However, no noticeable decrease in the overpotential for ORR was observed (Figure 5a). Apparently, the formation of Fe‐N_x_ sites in the catalyst is not dependent on the specific Fe ion source used during trans‐metalation. Additionally, we prepared another catalyst at a higher temperature of 1000 °C (FeNC‐LT‐1000), which displayed reduced activity with an *E*_1/2_ value of 0.78 V (Figure S7). It is well known that the pyrolysis temperature affects the overall nitrogen content in the final material and higher temperatures lead to a reduction of N sites, which is most likely the reason behind the lowered ORR activity.[^32^, ^59^, ^60^, ^61^]

**Figure 6:**
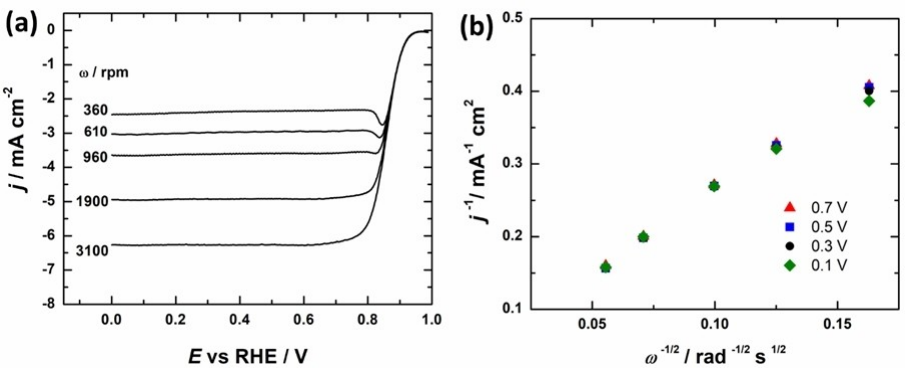
(a) RDE voltammetry curves for oxygen reduction in O_2_‐saturated 0.1 M KOH solution at different rotation rates for FeNC‐LT and (b) K−L plots derived from the RDE data.

However, it is likely that the density of the imprinting Mg sites formed during the first synthesis step would also lead to an increase in Fe‐N_x_ sites. As such, we introduced additional nitrogen sources (dicyandiamide and 1,10‐phenanthroline) to the precursor mixture, in order to increase the overall nitrogen content. Yet in both cases, the addition of other nitrogen sources negatively affected the electrocatalytic activity of the materials (Figure S7). We have previously shown that the added nitrogen precursor will react with the Mg precursor during the heat treatment forming MgCN_2_ nanoparticles. This likely negatively affects both the catalyst nanostructure and SD.^[50]^ In order to investigate the kinetics of the ORR in greater detail, the polarization curves were measured using different electrode rotation rates from 360 to 3100 rpm (Figure 6a). We observed well‐defined limiting current plateaus, with consistent onset potentials and varying limiting current densities. The Koutecky‐Levich (K−L) plots (Figure 6b) exhibit a good linearity indicating the first‐order reaction kinetics. The *n* value, calculated from the K−L slopes, reveals that the FeNC‐LT catalysts follow predominantly a 4‐electron pathway (Figure S8). The NC‐LT catalyst shows a strong potential dependency of the *n* value, with two distinct limiting current plateaus, favoring the 2e^−^ reduction pathway via HO_2_^−^ formation at more positive potentials and shifting more toward the 4e^−^ reduction pathway at higher overpotentials (Figure S8).

The accelerated stability testing of FeNC‐LT was conducted by continuous potential cycling in O_2_‐saturated 0.1 M KOH between 1.0 and 0.6 V vs RHE with a scan rate of 200 mV s^−1^ (Figure 5b). The ORR electrocatalytic activity was only slightly reduced after 10000 cycles (*ΔE*_1/2_=4.8 mV), even with a relatively low catalyst loading of 0.2 mg cm^−2^, indicating excellent stability in RDE tests

**Figure 7:**
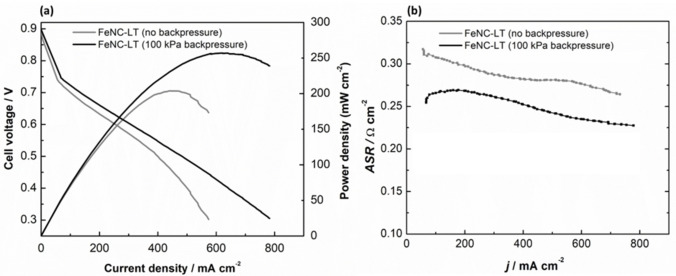
H_2_‐O_2_ AEMFC performance of FeNC‐LT cathode catalyst loaded to 0.89 mg cm^−2^ without backpressure and by applying backpressure of 100 kPa on both anode and cathode compartments. (a) Polarization and power density curves and (b) corresponding ASR values versus current density. The polarization curves were obtained by scanning from open circuit voltage at a scan rate of 2 mA sec^−1^.

The FeNC‐LT catalyst was further tested in single‐cell AEMFC measurements, where the catalysts displayed moderate activity as the cathode material with a peak power density (*P*_max_) value of 204 mW cm^−2^ (Figure 7a). Using a state‐of‐the‐art Pt/C cathode in an AEMFC comprising the same PtRu anode and AEM as used in this work, a *P*_max_ of ~1000 mW cm^−2^ was obtained (Figure S9a), on account of increased access to active sites (20x higher metal content in the Pt catalyst). The higher activity of the Pt/C cathode AEMFC is further evidenced by comparing the catalytic region performance. For instance, at 0.8 V, the current density of the FeNC‐LT AEMFC is 29 mA cm^−2^, as compared to 313 mA cm^−2^ for the Pt/C AEMFC. Compared to the thin catalyst layers of PGM‐based catalysts, the catalyst layer prepared with the FeNC‐LT cathode is significantly thicker resulting from the low packing factor (∼100 μm mg^−1^ cm^−2^), consequently, the area specific resistance (ASR) for this AEMFC is quite high (see Figure 7b). As shown in Figure S9b, the ASR of the Pt/C cathode AEMFC is ~3x lower than that of the FeNC‐LT AEMFC. This can be attributed to the almost 4x thicker cathode catalyst layer of 128 μm (FeNC‐LT) compared to 32 μm (Pt/C).

Applying backpressure of 100 kPa somewhat alleviated this factor by lowering the ASR, and a *P*_max_ value of 256 mW cm^−2^ was obtained for FeNC‐LT. However, optimizing the synthesis conditions of the carbon‐based catalysts together with the catalyst layer design, is likely necessary to obtain higher performance in AEMFCs.

## Conclusions

This study demonstrates the potential of the ionothermal active‐site imprinting approach, achieved by utilizing Mg(NO_3_)_2_ and Honeyol as the precursors. This method enables the separation of carbon formation from active site creation, overcoming the limitations of existing synthetic methods, while creating a hierarchically porous structure. We showed that Mg(NO_3_)_2_ can be used to imprint Mg‐N_x_ sites that can subsequently undergo trans‐metalation with Fe, and simultaneously produce a hierarchically porous carbon structure, through the in‐situ formation of MgO nanoparticles that serve as the sacrificial template. The best‐performing electrocatalyst (FeNC‐LT) revealed excellent electrocatalytic activity for ORR in alkaline conditions (*E*_1/2_=0.87 V), similar to that of the commercial Pt/C catalyst.

## Experimental

### Preparation of Electrocatalysts

The catalyst materials were produced using Honeyol^TM^, a carbon precursor consisting of resorcinol derivatives with a 5‐methylresorcinol content of at least 48 wt % (VKG Oil AS, Estonia). Honeyol was mixed with Mg(NO_3_)_2_ ⋅ 6H_2_O (98 %, Alfa Aesar) using a pestle and mortar, with a mass ratio of Honeyol‐to‐Mg(NO_3_)_2_ of 1 : 1. For comparison a material with a mass ratio of 1 : 2 was also prepared (Table S1). The well‐homogenized mixture was then transferred into a ceramic boat and pyrolyzed in a nitrogen flow at 800 °C for 1 h with a ramp rate of 10 °C min^−1^. During the pyrolysis the Mg(NO_3_)_2_ ⋅ 6H_2_O acts as a solvent, porogen and nitrogen dopant. The in‐situ formed MgO nanoparticles act as the sacrificial template and are removed after the pyrolysis by dispersing the resulting MgO@NC‐LT material in a 1 M HCl (Sigma‐Aldrich) solution for 2 h. The obtained carbon material (NC‐LT) was thoroughly washed with Milli‐Q water, filtered and dried overnight in an oven at 60 °C, followed by grinding. As a comparison, a higher pyrolysis temperature of 1000 °C was used to prepare the material NC‐LT‐1000. In addition, two different nitrogen precursors were added in the initial mixing stage to Honeyol and Mg(NO_3_)_2_ ⋅ 6H_2_O, in order to increase the overall nitrogen content: dicyandiamide (DCDA, Sigma‐Aldrich) and 1,10‐phenanthroline (PHEN, Acros Organics) with a mass ratio of Honeyol‐to‐N‐precursor of 0.2 and 14.2, respectively. These materials are denoted as NC‐LT‐DCDA and NC‐LT‐PHEN.

In order to obtain the ORR‐active electrocatalysts, an ion‐exchange procedure was used to replace Mg‐N_x_ sites with Fe‐N_x_, following the method proposed by Fellinger and co‐workers.^[34]^ The materials were dispersed in methanol, to which a certain amount of FeCl_2_ ⋅ 4H_2_O (97 %, Sigma‐Aldrich) or FeCl_3_ (98 %, Thermo Scientific) was added to obtain a concentration of 2.5×10^−2^ M. The resulting mixture was refluxed under constant stirring for 24 h. The catalyst materials were then washed with Milli‐Q water and dispersed in a 0.5 M H_2_SO_4_ solution overnight. The materials were then washed with water, filtered, dried in an oven and pyrolyzed at 800 °C for 1 h with a ramp rate of 50 °C min^−1^, to obtain the final catalysts denoted as indicated in Table S1.

### Physical Characterization Methods

The materials were characterized by scanning electron microscopy (SEM), high‐resolution scanning transmission electron microscopy (HR‐S‐TEM), Raman spectroscopy, nitrogen adsorption/desorption measurements, X‐ray photoelectron spectroscopy (XPS), X‐ray diffraction (XRD) measurements and microwave plasma atomic emission spectroscopy (MP‐AES). A detailed description of the physical characterization of the materials is given in the Supplementary Material.

### Electrochemical Characterization Methods

The rotating disk electrode (RDE) method was used to study the ORR activity of the catalysts. The catalyst inks were prepared by ultrasonically dispersing the catalyst materials The GC electrode was drop‐casted with the ink until a catalyst loading of 200 μg cm^−2^ was obtained. The ORR measurements were performed in 0.1 M KOH aqueous solution saturated with O_2_ (99.999 %, Linde) at various electrode rotation rates (*ω*) between 360–3100 rpm. Durability tests were performed by cycling the electrode in the potential range from 1.0 to 0.6 V vs. RHE at 200 mV s^−1^, while RDE polarization curves were recorded after 1000, 5000, 10000, and 15000 potential cycles.

In order to evaluate the FeNC‐LT catalyst′s performance as a cathode in an AEMFC, gas diffusion electrodes (GDEs) with active areas sizes of 5 cm^2^ were prepared. To prepare the inks, a powdered anion‐exchange ionomer (AEI), a cross‐linked polystyrene compound functionalized with trimethylamine (Fumatech), along with FeNC‐LT and ~10 mL of total solvents (deionized water and 2‐propanol) were added to the mortar ground followed by sonication. For comparison, a Pt/C cathode was created using Alfa Aesar, 40 % Pt on carbon black HiSPEC 4000. A PtRu/C catalyst (Alfa Aesar, 40 % Pt and 20 % Ru on carbon black HiSPEC 10000) was employed as the catalyst to make the anode. Following sonication, the inks were sprayed directly onto a 5 cm^2^ gas diffusion layers. For cell assembly cell assembly, the cathode and anode GDEs and a FAA‐3‐05‐RF AEM were pressed between two 5 cm^2^ single‐serpentine graphite bipolar flow field plates with Teflon gaskets.The cell was tested in a Scribner Associates 850 E Fuel Cell test station under H_2_/O_2_ gas flows. A more detailed description is given in the Supporting Information.

## Conflict of Interests

The authors declare no conflict of interest.

1

## Supporting information

As a service to our authors and readers, this journal provides supporting information supplied by the authors. Such materials are peer reviewed and may be re‐organized for online delivery, but are not copy‐edited or typeset. Technical support issues arising from supporting information (other than missing files) should be addressed to the authors.

Supporting Information

## Data Availability

The data that support the findings of this study are available in the supplementary material of this article.
