# Supplementary material for: Hierarchically Porous Fe−N−C Single‐Atom Catalysts via Ionothermal Synthesis for Oxygen Reduction Reaction
Source: ChemSusChem. 2024 Oct 23;18(2):e202401332. doi: 10.1002/cssc.202401332 (PMC11739835; doi:10.1002/cssc.202401332)
Supplement: Supplementary file 1 — Supporting Information [file CSSC-18-e202401332-s001.pdf]

ChemSusChem

Supporting Information

Hierarchically Porous Fe–N–C Single-Atom Catalysts via Ionothermal Synthesis for Oxygen Reduction Reaction

Kaarel Kisand, Ave Sarapuu, John C. Douglin, Arvo Kikas, Maike Käärik, Jekaterina Kozlova, Jaan Aruväli, Alexey Treshchalov, Jaan Leis, Vambola Kisand, Kaupo Kukli, Dario R. Dekel,* and Kaido Tammeveski*

Supporting Information

Hierarchically Porous Fe-N-C Single-atom Catalysts via Ionothermal Synthesis for Oxygen Reduction Reaction

Kaarel Kisand[a], Ave Sarapuu[a], John C. Douglin[d], Arvo Kikas[b], Maike Käärik[a], Jekaterina Kozlova[b], Jaan Aruväli[c], Alexey Treshchalov[b], Jaan Leis[a], Vambola Kisand[b], Kaupo Kukli[b], Dario R. Dekel[d,e*], and Kaido Tammeveski[a,*]

[a]*Institute of Chemistry, University of Tartu, Ravila 14a, 50411 Tartu, Estonia*

[b]*Institute of Physics, University of Tartu, W. Ostwald Str. 1, 50411 Tartu, Estonia*

[c]*Institute of Ecology and Earth Sciences, University of Tartu, Vanemuise 46, 51014 Tartu, Estonia*

[d]*The Wolfson Department of Chemical Engineering, Technion—Israel Institute of Technology, 3200003 Haifa, Israel*

[e]*The Nancy & Stephen Grand Technion Energy Program (GTEP), Technion – Israel Institute of Technology, 3200003, Haifa, Israel*

[*]Corresponding authors. *E-mail addresses:* dario@technion.ac.il (D. R. Dekel), kaido.tammeveski@ut.ee (K. Tammeveski)

Experimental

Table S1. The precursors and conditions used in the preparation of the catalyst materials.

Catalyst	Honeyol (g)	$Mg(NO_3)_2{\cdot}6H_2O$ (g)	Metal source	Additive	Pyrolysis temperature (°C)
NC-LT	1	1	-	-	800
FeNC-LT	1	1	$FeCl_2{\cdot}4H_2O$	-	800
FeNC-LT2	1	2	$FeCl_2{\cdot}4H_2O$	-	800
FeNC-LT-$FeCl_3$	1	1	$FeCl_3$	-	800
FeNC-LT-1000	1	1	$FeCl_2{\cdot}4H_2O$	-	1000
FeNC-LT-DCDA	1	1	$FeCl_2{\cdot}4H_2O$	DCDA	800
FeNC-LT-PHEN	1	1	$FeCl_2{\cdot}4H_2O$	PHEN	800

Physical characterization methods

The scanning electron microscopy (SEM) samples were prepared by drop-casting a suspension of the catalyst material onto polished GC disks. The SEM analysis was conducted with Helios NanoLab^{TM} 600 (FEI) microscope. The energy dispersive X-ray spectroscopy (EDX) detector INCA Energy 350 (Oxford Instruments) mounted on the microscope was used for elemental characterization.

For the high-resolution scanning transmission electron microscopy (HR-S-TEM) the samples were deposited onto Lacey carbon supports on Cu 300 mesh grids (Agar Scientific). The TEM measurements were made with Titan Themis 200 (FEI) microscope with an accelerating voltage of 200 kV in scanning mode with both bright field (BF) and high-angle annular dark field (HAADF) detectors. Elemental mapping was taken from the same area as HAADF images with Super-X EDX analyzer ChemiSTEM (FEI/Bruker).

For obtaining the N_2 adsorption/desorption isotherms, the materials were vacuum-dried for 6 h at 150 °C before the measurement. The measurement was done using NovaTouch LX2 instrument (Quantachrome) at the boiling point of nitrogen (77 K). The total pore volume (V_{tot}) was measured at the saturation pressure of N_2 (P/P_0 = 0.97). Pore size distribution (PSD) was calculated using a quenched solid density functional theory (QSDFT) equilibria model for slit-cylindrical-spherical type pores. All of the calculations were done using TouchWin 1.11 software (Quantachrome Instruments).

The X-ray photoelectron spectroscopy (XPS) analysis was conducted at ultra-high vacuum conditions using a non-monochromatic twin anode X-ray tube (Thermo XR3E2) with the characteristic energy of 1253.6 eV (Mg K_α) and an electron energy analyzer SCIENTA SES 100. The survey scan was measured in the energy range from 900 to 0 eV, pass energy of 200 eV, step size of 0.5 eV, step duration 0.2 s and number of scans 5. Detailed XPS spectra were collected with a step size of 0.1 eV and step duration of 0.2 s, with the number of scans being at least 25. The data was processed with CasaXPS (version 2.3.17), during which the K_α and K_β satellites were removed. Gauss–Lorentz hybrid function (GL 70, Gauss 30 %, Lorentz 70 %) and a blend of linear and Shirley-type backgrounds were used for peak fitting.

X-ray diffraction (XRD) measurement was performed on Bruker D8 Advance diffractometer with Ni-filtered Cu Kα radiation. The diffraction patterns were obtained with scanning steps of 0.0126° 2θ in the range from 5° to 89° and the counting time of 525 s per step. The data was analyzed with Topas 6 (Bruker) software.

The transition metal concentration (Ni, Fe and Co) in the samples was determined by microwave plasma atomic emission spectroscopy (MP-AES) using Agilent 4210 MP-AES. 10 mg of the catalyst material was dissolved with Anton Paar Multiwave PRO microwave digestion system in NXF100 vessels (PTFE/TFM liner) in a mixture of 4 mL of HNO_3 (65%, Carl Roth, ROTHIPURAN® Supra) and 2 mL of H_2O_2 (30%, Carl Roth, ROTHIPURAN®). The dissolved samples were digested at 230 °C and at pressures between 45-50 bar. After the dissolution process, the samples were diluted with 2% HNO_3 solution to obtain metal concentrations of around 5mg/L and measured.

Micro-Raman spectra were recorded in the back-scattering geometry on an inVia Renishaw spectrometer in conjugation with a confocal microscope (Leica Microsystems CMS GmbH, Germany), 50x objective and an argon ion laser operated at 514.5 nm. All samples were suspended in water and drop-coated onto silicon substrates. To avoid thermal decomposition of the sample, the laser power density was minimized by decreasing the laser power and defocusing the laser spot to about 15 μm. The Raman spectra were normalized to the intensity of the G band. The spectra were fitted following the five-peak model where G peak (~1580 cm^{-1}) corresponds to the ideal graphitic lattice; D1 (~1350 cm^{-1}) to disordered graphitic lattice-graphene layer edge; D2 (~1610 cm^{-1}) to disordered graphitic lattice; D3 (~1500 cm^{-1}) to amorphous carbon and D4 (~1200 cm^{-1}) to disordered graphitic lattice. The Peak Analyser software in OriginPro 9 was used for Raman spectra processing.

Electrochemical measurements

The catalyst inks were prepared by ultrasonically dispersing the catalyst materials in a mixture of H_2O and 2-propanol (1:1) by volume and Nafion ionomer solution (5 wt.% solution in lower alcohols, Aldrich) with ionomer-to-carbon (I/C) ratio of 0.23, resulting in catalyst powder content of 2 mg ml^{-1} in suspensions. Glassy carbon (GC) electrode (GC20-SS, Tokai Carbon, geometric surface area of 0.196 cm^2) was polished on 1 and 0.3 μm alumina (Buehler) slurries. The GC electrode was drop-casted with the ink until a catalyst loading of 200 μg cm^{-2} was obtained. The same procedure was used to prepare GC electrodes coated with a commercial Pt/C catalyst (20 wt.%, E-TEK).

The rotating disk electrode (RDE) method was used to study the ORR activity of the catalysts. The electrochemical measurements were performed in a conventional three-electrode glass cell with an Autolab potentiostat/galvanostat PGSTAT30 (Metrohm-Autolab, The Netherlands). Potentials were measured against a saturated calomel electrode (SCE) and a carbon rod was used as the counter electrode. Various electrode rotation rates (*w*) between 360-3100 rpm were used, controlled by a CTV101 speed control unit, which was connected to an EDI101 rotator (Radiometer). The ORR measurements were performed in 0.1 M KOH aqueous solution saturated with O_2 (99.999%, Linde). The background current was recorded in Ar-saturated (99.999%, Linde) 0.1 M KOH solution and subtracted from the experimental O_2 reduction current. The measured values of the potentials were converted to reversible hydrogen electrode (RHE) scale as follows: $E_{RHE} = E_{SCE} + 1.008$ V. Electrochemical impedance spectroscopy (EIS) was performed with the catalyst-coated GC electrodes in O_2-saturated 0.1 M KOH to calculate

the solution resistance, and iR correction was applied to the ORR polarization data with Nova 2.1 software.

Durability tests were performed by cycling the electrode in the potential range from 1.0 to 0.6 V vs. RHE at 200 mV s^{-1}, while RDE polarization curves were recorded after 1000, 5000, 10000, and 15000 potential cycles.

The RDE data was analyzed using the Koutecky-Levich (K-L) equation:[2]

$$\frac{1}{j} = \frac{1}{j_k} + \frac{1}{j_d} = \frac{1}{j_k} - \frac{1}{0.62nFD_{O_2}^{\frac{2}{3}}\nu^{-\frac{1}{6}}\omega^{\frac{1}{2}}c_{O_2}^{b}} \quad (1)$$

where j is the measured current density, j_k and j_d are the kinetic and diffusion-limited current densities, respectively, n is the number of electrons transferred per O_2 molecule, F is the Faraday constant (96 485 C mol^{-1}), ω is the rotation rate (rad s^{-1}), D_{O2} is the diffusion coefficient of oxygen (1.9×10^{-5} cm^2 s^{-1}), C_{O2} is the concentration of oxygen in the bulk (1.2×10^{-6} mol cm^{-3}), and ν is the kinematic viscosity of the electrolyte solution (0.01 cm^2 s^{-1}).

Membrane electrode assembly construction and fuel cell tests

In order to evaluate the FeNC-LT catalyst’s performance as a cathode in an AEMFC, gas diffusion electrodes (GDEs) with active areas sizes of 5 cm^2 were made following similar procedures outlined in our earlier work.[3–6] To prepare the cathode, 10 mg of powdered anion-exchange ionomer (AEI), a cross-linked polystyrene compound functionalized with trimethylamine (Fumatech), was first added to a mortar and ground for 3 minutes. Following that, 25 mg of FeNC-LT and ~10 mL of total solvents (1 mL of deionized water and up to 9 mL of 2-propanol) were added to the mortar containing the AEI, mixed and ground for an additional 10 min to create a low viscosity catalyst ink. For comparison, a Pt/C cathode was created using Alfa Aesar, 40% Pt on carbon black HiSPEC 4000 following similar procedures and loaded to 0.8 mg_{Pt} cm^{-2} [7–9] A PtRu/C catalyst (Alfa Aesar, 40% Pt and 20% Ru on carbon black HiSPEC 10000) was employed as the catalyst to make the anode. Approximately 19 mg of AEI, 51 mg of PtRu/C and an additional 25 mg of carbon black (Vulcan XC-72) were added to the mortar and ground in a 1:9 mL deionized water-to-2-propanol solvent ratio to create the ink.

Both FeNC-LT, Pt/C and PtRu/C catalyst inks were transferred to individual 50 mL plastic centrifuge tubes and sonicated in an ice-cooled Grant XUBA3 ultrasonic bath at 100% intensity for one hour. Following sonication, the cathode inks were sprayed directly onto a 5 cm^2 gas diffusion layers (FuelCellStore, Toray Carbon Paper 060 with Micro Porous Layer) with an

Iwata HP-TH professional airbrush and dried at 140 °C on a drying plate. The final loading of the FeNC-LT GDE was 0.89 $mg_{FeNC-LT}$ cm^{-2} and the Pt/C was 0.8 mg_{Pt} cm^{-2}. The anode ink was similarly spray deposited onto a 5 cm^2 gas diffusion layer (FuelCellStore, Toray Carbon paper 060-TGP-H-060 with 20 wt % PTFE wet proof) to a PGM loading of 0.75 mg_{PtRu} cm^{-2}. Before cell assembly, the two GDEs and a 9 cm^2 piece of the FAA-3-05-RF AEM (polymer reinforced with porous ePTFE-film, 7 µm average fully hydrated thickness) were submerged in separate petri dishes containing 1 M KOH aqueous solution. The solution was changed every 20 min to ensure complete ion-exchange. Following the soaking process, the AEMFCs were assembled between two 5 cm^2 single-serpentine graphite bipolar flow field plates with Teflon gaskets and torqued to 4.5 N m to achieve an average gas diffusion layer compression of 35%. The thicknesses of the anode, cathode and AEM were measured with a Mitutoyo ABS Digital Thickness Gauge (Item number: 547-526S) prior to cell assembly to determine the appropriate gaskets. The cell was tested in a Scribner Associates 850E Fuel Cell test station under H_2/O_2 gas flows of 0.5 SLPM at a cell temperature of 60 °C and anode and cathode dewpoints set at 55 and 57 °C, respectively.

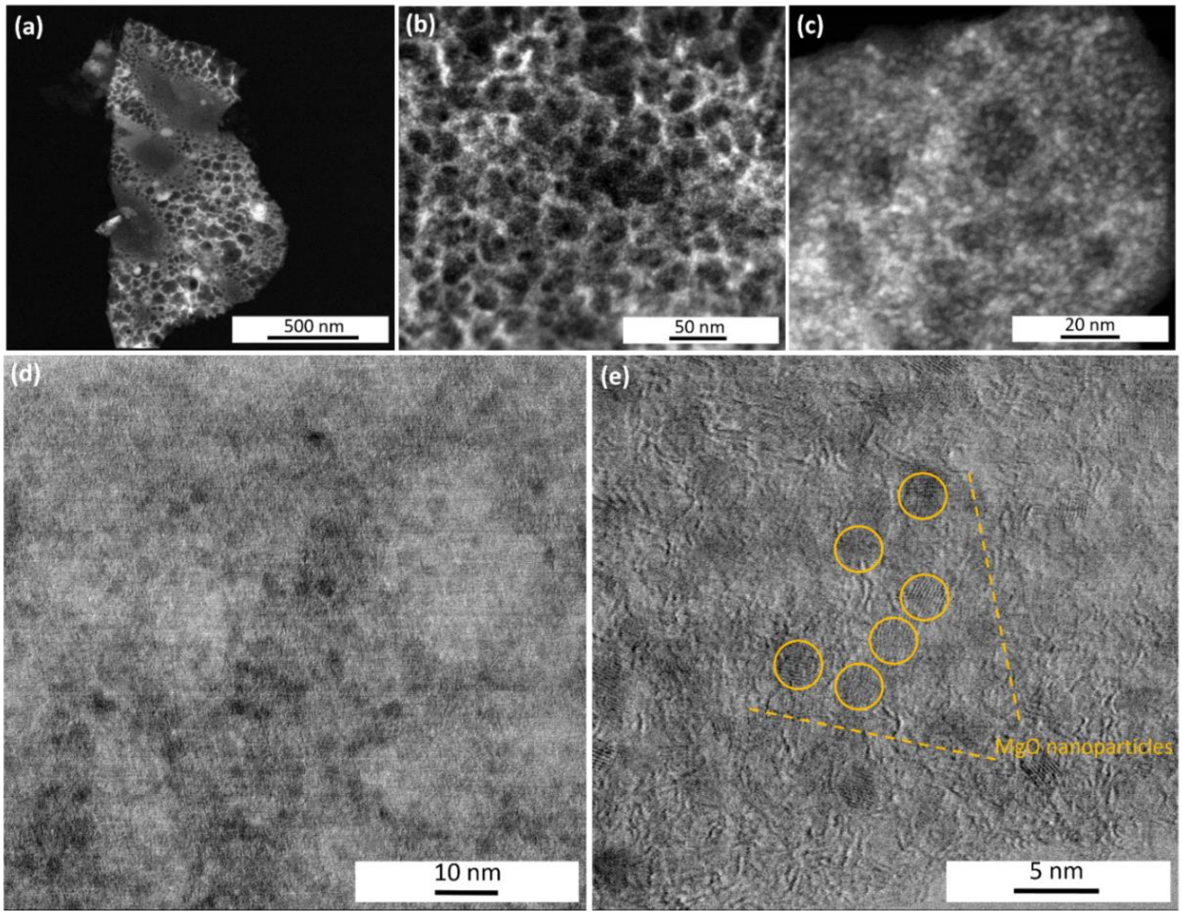

Figure S1. (a, b, c) HAADF-STEM images and (d, e) BF-STEM images of MgO@NC-LT material.

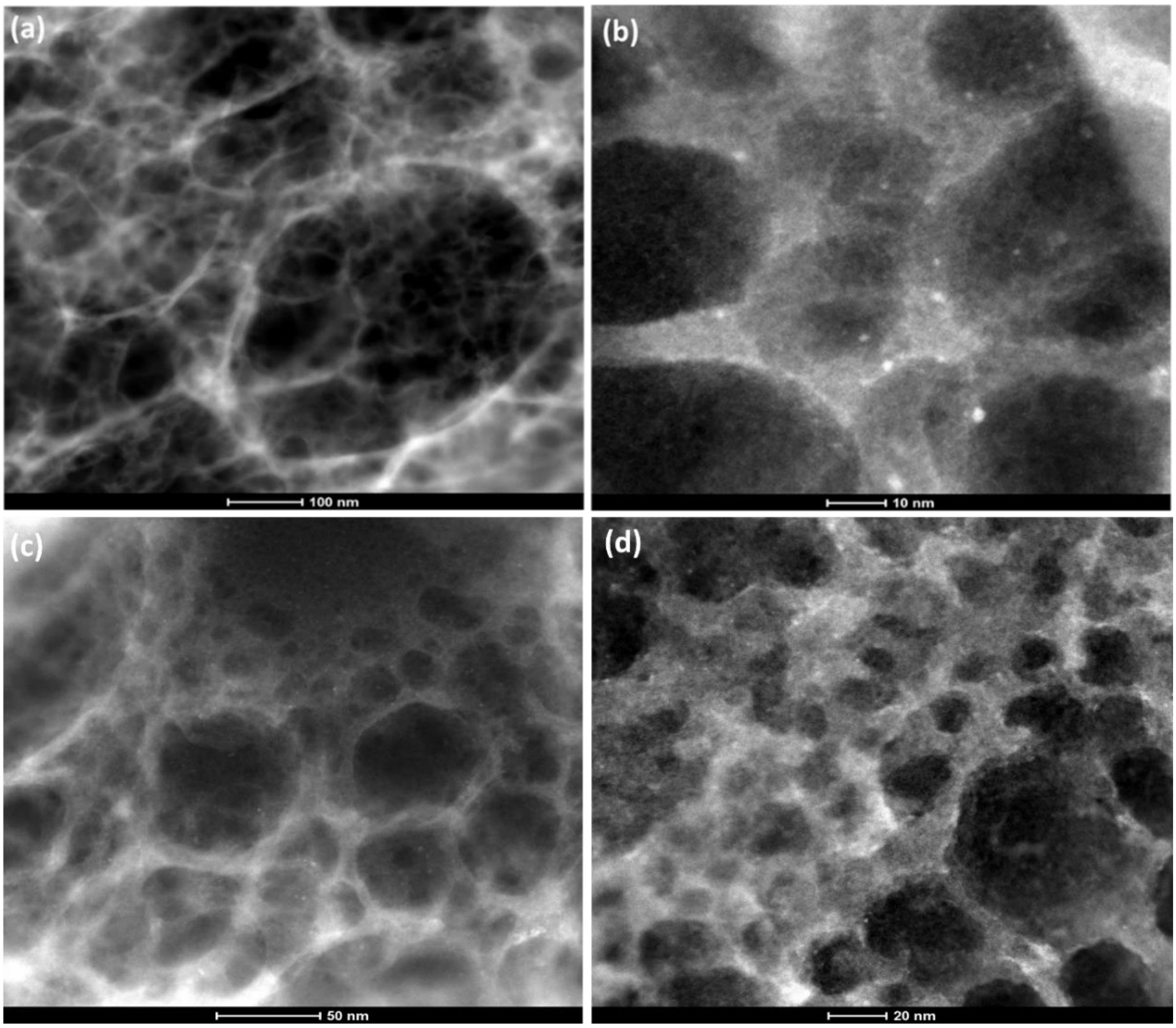

Figure S2. HAADF-STEM images of FeNC-LT material displaying (a, c and d) hierarchical porosity and (b) metallic clusters.

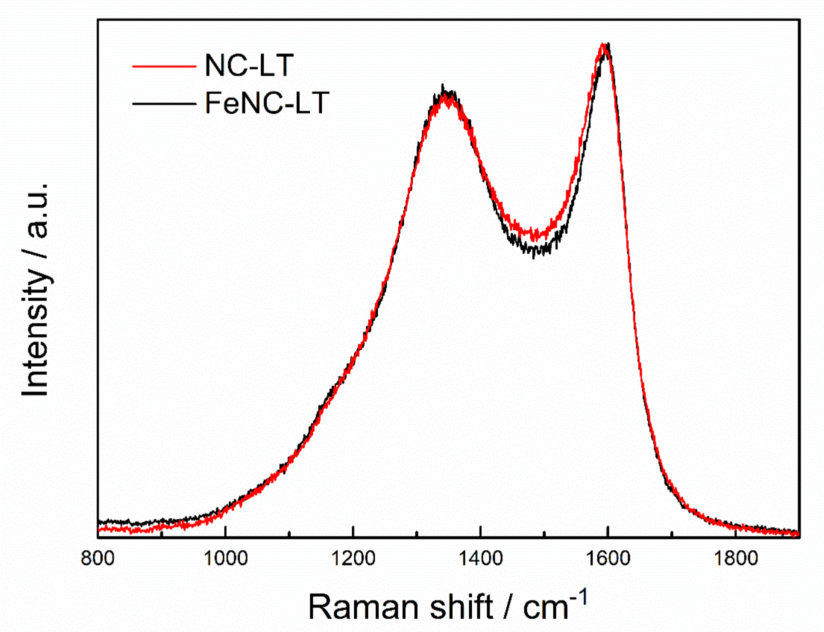

Figure S3. Raman spectra of the catalyst materials.

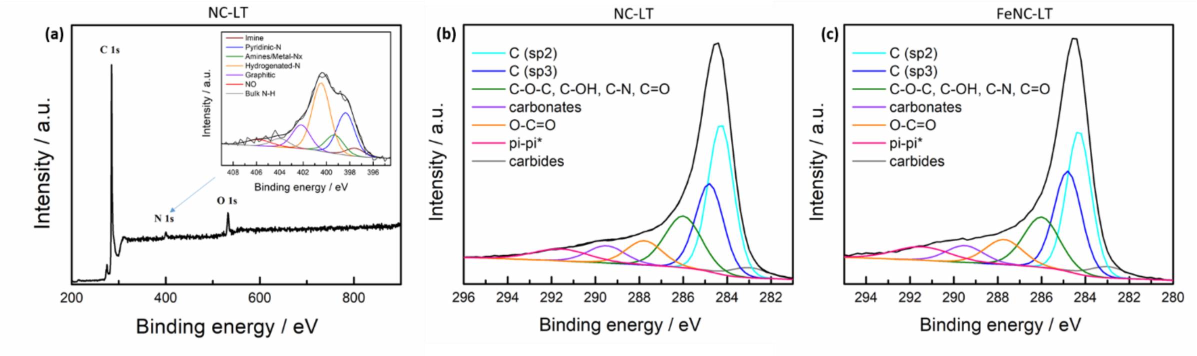

Figure S4. (a) XPS survey spectra for NC-LT with the inset showing high-resolution N 1s XPS spectra and (b, c) C 1s high-resolution XPS spectra of the catalyst materials.

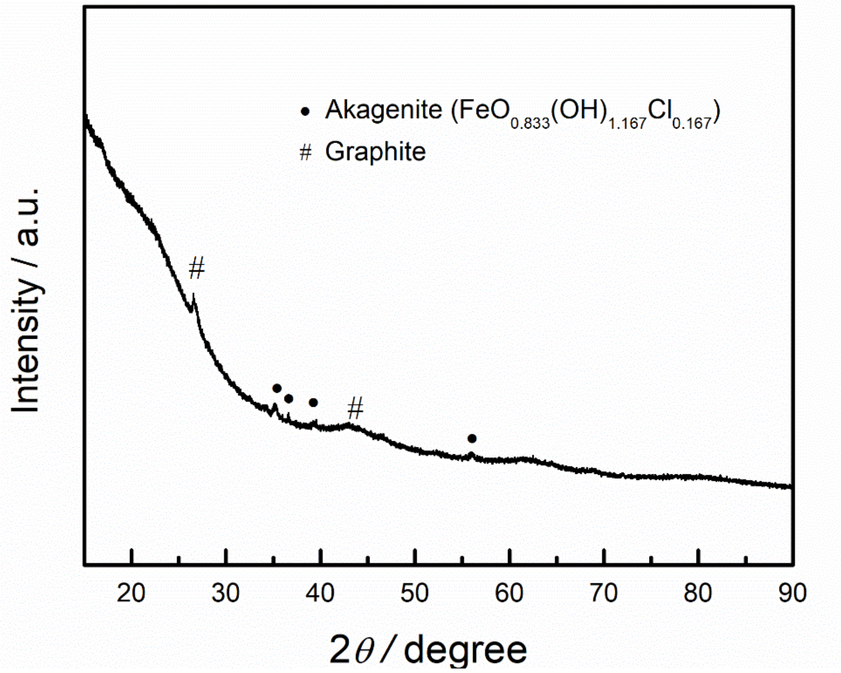

Figure S5. XRD pattern of the FeNC-LT material after acid-washing, before the 2nd pyrolysis.

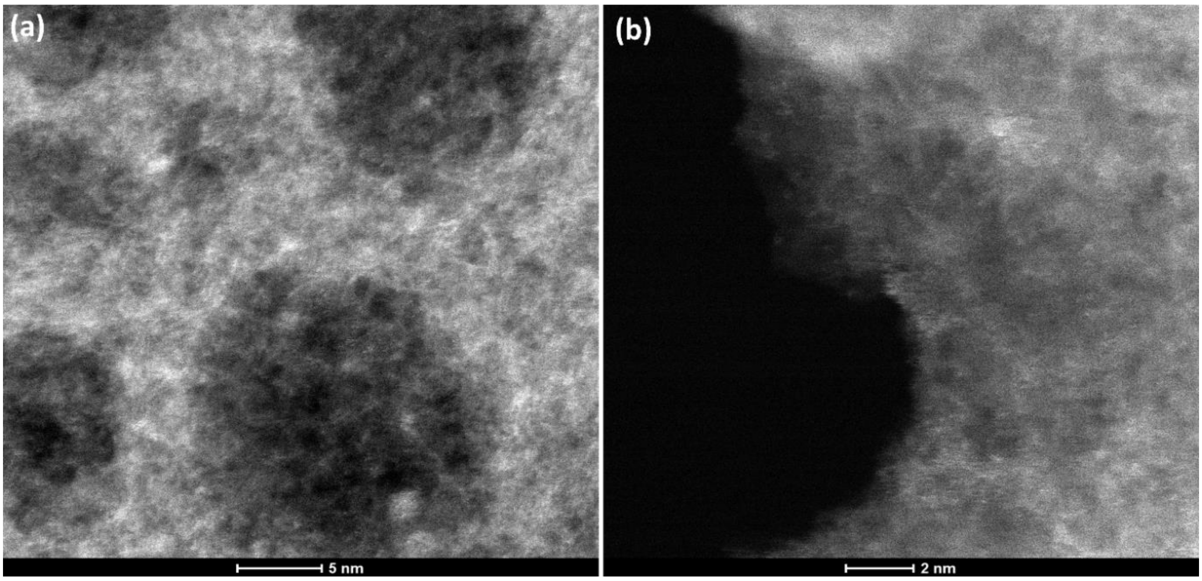

Figure S6. HAADF-STEM images of FeNC-LT catalyst displaying the atomically dispersed sites.

Table S2. Elemental composition of the catalysts obtained from SEM-EDX analysis and bulk metal composition obtained with MP-AES.

Catalyst	Elemental composition by SEM-EDX (wt.%)					Bulk metal content by MP-AES (wt.%)	
	C	N	O	Mg	Fe	Mg	Fe
NC-LT	84.0	4.9	8.9	1.2	-	2.18	-
FeNC-LT	84.3	4.0	7.4	1.4	2.0	2.26	2.1

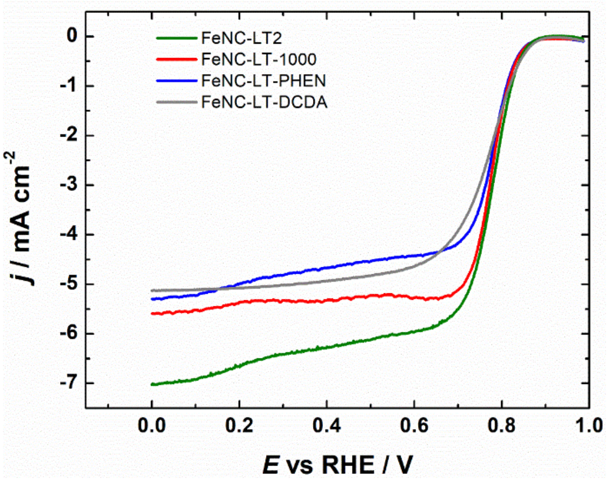

Figure S7. Comparison of RDE voltammetry curves for ORR on various PGM-free catalysts and in O_2-saturated 0.1 M KOH solution at 1900 rpm.

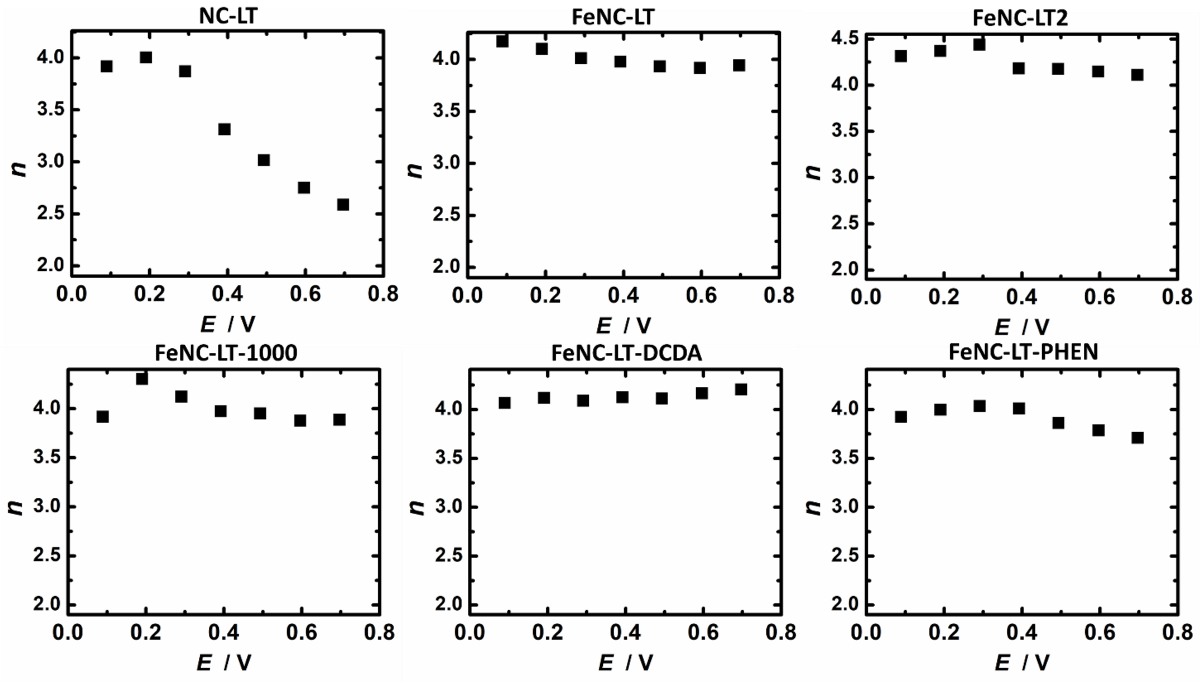

Figure S8. Dependence of *n* on the electrode potential.

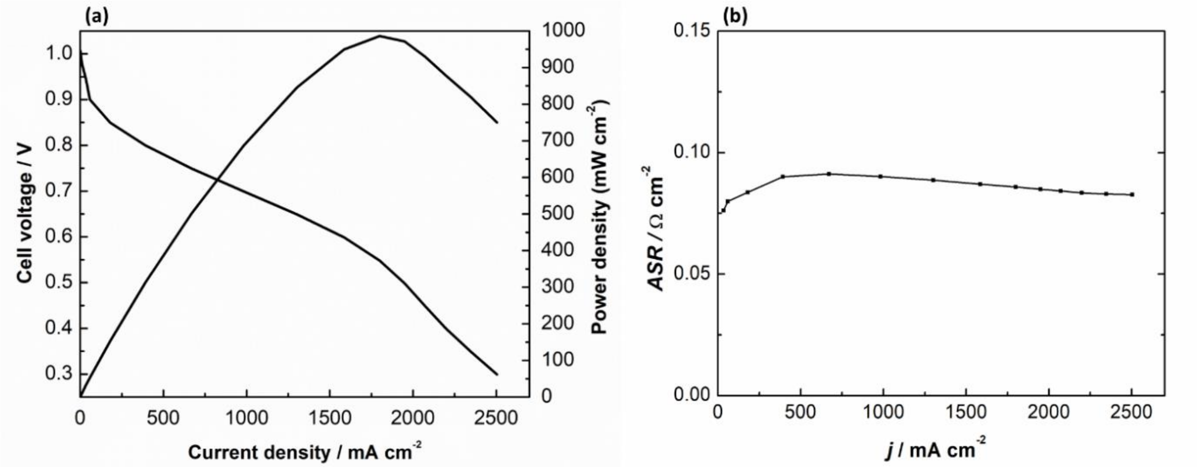

Figure S9. H_2-O_2 AEMFC performance of Pt/C cathode catalyst loaded to 0.8 mg cm^{-2} without backpressure. (a) Polarization and power density curves and (b) corresponding ASR values versus current density.

Refrences

[1] A. Mehmood, J. Pampel, G. Ali, H.Y. Ha, F. Ruiz-Zepeda, T.-P. Fellinger, Facile Metal Coordination of Active Site Imprinted Nitrogen Doped Carbons for the Conservative Preparation of Non-Noble Metal Oxygen Reduction Electrocatalysts, Adv. Energy Mater. 8 (2018) 1701771. https://doi.org/10.1002/aenm.201701771.

[2] A.J. Bard, L.R. Faulkner, Electrochemical Methods: Fundamentals and Applications, 2nd ed., New York: Wiley, 2001.

[3] W. Zhu, Y. Pei, J.C. Douglin, J. Zhang, H. Zhao, J. Xue, Q. Wang, R. Li, Y. Qin, Y. Yin, D.R. Dekel, M.D. Guiver, Multi-scale study on bifunctional Co/Fe–N–C cathode catalyst layers with high active site density for the oxygen reduction reaction, Appl. Catal. B: Environ. 299 (2021) 120656. https://doi.org/10.1016/j.apcatb.2021.120656.

[4] J.C. Douglin, R.K. Singh, S. Haj-Bsoul, S. Li, J. Biemolt, N. Yan, J.R. Varcoe, G. Rothenberg, D.R. Dekel, A high-temperature anion-exchange membrane fuel cell with a critical raw material-free cathode, Chem. Eng. J. Adv. 8 (2021) 100153. https://doi.org/10.1016/j.ceja.2021.100153.

[5] J.C. Douglin, R.K. Singh, E.R. Hamo, M.B. Hassine, P.J. Ferreira, B.A. Rosen, H.A. Miller, G. Rothenberg, D.R. Dekel, Performance optimization of PGM and PGM-free catalysts in anion-exchange membrane fuel cells, J. Solid State Electrochem. 26 (2022) 2049–2057. https://doi.org/10.1007/s10008-022-05261-4.

[6] R.K. Singh, J.C. Douglin, L. Jiang, K. Yassin, S. Brandon, D.R. Dekel, CoOx-Fe3O4/N-rGO Oxygen Reduction Catalyst for Anion-Exchange Membrane Fuel Cells, Energies 16 (2023) 3425. https://doi.org/10.3390/en16083425.

[7] J.C. Douglin, K. Vijaya Sankar, A.L.G. Biancolli, E.I. Santiago, Y. Tsur, D.R. Dekel, Quantifying the Resistive Losses of the Catalytic Layers in Anion-Exchange Membrane Fuel Cells, ChemSusChem 16 (2023) e202301080. https://doi.org/10.1002/cssc.202301080.

[8] A.L.G. Biancolli, S. Bsoul-Haj, J.C. Douglin, A.S. Barbosa, R.R. de Sousa, O. Rodrigues, A.J.C. Lanfredi, D.R. Dekel, E.I. Santiago, High-performance radiation grafted anion-exchange membranes for fuel cell applications: Effects of irradiation conditions on ETFE-based membranes properties, J. Membrane Sci. 641 (2022) 119879. https://doi.org/10.1016/j.memsci.2021.119879.

[9] J.C. Douglin, J.R. Varcoe, D.R. Dekel, A high-temperature anion-exchange membrane fuel cell, J. Power Sources Adv. 5 (2020) 100023. https://doi.org/10.1016/j.powera.2020.100023.
